# β-caryophyllene sensitizes hepatocellular carcinoma cells to chemotherapeutics and inhibits cell malignancy through targeting MAPK signaling pathway

**DOI:** 10.3389/fphar.2024.1492670

**Published:** 2024-12-13

**Authors:** Irum Basheer, Hai Wang, Guangyue Li, Shah Jehan, Ali Raza, Chentao Du, Najeeb Ullah, Dangdang Li, Guangchao Sui

**Affiliations:** ^1^ Key Laboratory of Saline-Alkali Vegetation Ecology Restoration, Ministry of Education, College of Life Science, Northeast Forestry University, Harbin, China; ^2^ Intelligent Biomedical Labs, Institute for Advanced Study, Hangzhou, Zhejiang, China; ^3^ Department of Vascular Surgery, The First Affiliated Hospital of Guangzhou Medical University, Guangzhou, Guangdong, China; ^4^ College of Wildlife and Protected Area, Northeast Forestry University, Harbin, China

**Keywords:** hepatocellular carcinoma, β-caryophyllene, anti-cancer activity, combinatorial treatment, RNA-seq, placental growth factor (PGF), MAPK signaling pathway

## Abstract

**Background:**

β-caryophyllene (BCP) is a naturally occurring bicyclic sesquiterpene extracted from various plants, and widely used as a medicinal agent for various diseases. During hepatocellular carcinoma (HCC) development, cancer cells generally exhibit increased cell proliferation due to mutations or aberrant expression of key regulatory genes. The current study determines the cytotoxic effects of BCP alone or in combination with doxorubicin (DOX) and cisplatin (DDP) on HCC cells, and elucidates the underlying mechanism of BCP to exert its anticancer activities.

**Materials and methods:**

HepG2, SMMC-7721 HCC cells, and HL-7702 normal liver cells were treated with BCP, DOX, and DDP individually or combinatorially. Cell proliferation assay, flow cytometric assay, and Western blot were employed to evaluate the cytotoxic effects of these treatments. Transwell assays were used to examine BCP’s effects on HCC cell migration and invasion. RNA-seq analysis was used to determine BCP’s primary target genes in HepG2 cells. Integrative analysis of differentially expressed genes (DEGs) of RNA-seq data with an HCC TCGA dataset identified BCP-targeted genes that were verified by RT-qPCR analysis. Ectopic gene expression, cell viability, and colony formation assay were performed to validate the primary targets of BCP.

**Results:**

BCP selectively inhibited HCC cell proliferation while exhibited relatively low toxicity in normal liver cells; however, DOX and DDP showed higher toxicity in normal cells than that in HCC cells. In combinatorial treatments, BCP synergistically enhanced cytotoxicity of DOX and DDP in HCC cells but this effect was markedly reduced in HL-7702 cells. BCP treatment reduced migration and invasion of HCC cells. Furthermore, RNA-seq analyses of BCP-treated HepG2 cells identified 433 protein-coding DEGs. Integrative analyses revealed five BCP-targeted DEGs regulating the MAPK signaling pathway. Among these five genes, three displayed a significantly positive correlation of their expression with the overall survival of HCC patients. As a primary target, PGF was significantly downregulated by BCP treatment, and its exogenous expression desensitized HCC cells to BCP-mediated inhibition.

**Discussion:**

BCP inhibits malignant properties of HCC and synergistically sensitizes the anticancer activity of DOX and DDP. In HCC cells, BCP primarily targets the PGF gene and MAPK signaling pathway.

## 1 Introduction

Hepatocellular carcinoma (HCC) is the most common primary liver malignancy and the sixth most common cancer worldwide, with climbing incidence rates over the last 3 decades. Approximately over 1 million of new HCC cases will be diagnosed annually by 2025 (Llovet et al., 2016; Konyn et al., 2021). The occurrence of HCC is generally associated with hepatitis and cirrhosis, accounting for more than 80%–90% of the cases. Cirrhosis, regardless of its cause, increases 7% risk of HCC development (Ringelhan et al., 2018; Niture et al., 2021). The current HCC treatment approaches include surgical resection, liver transplantation, ablation treatments, and systematic chemotherapies. These therapeutic options depend upon overall health status of the patients (Renne et al., 2021). Among these therapies, surgical resection and liver transplantation are the first potential curative treatments for early-stage liver cancer patients and correlate with the most promising outcomes, with a 5-year survival of 70%–80%. However, only 15% of patients are suitable to receive these treatments, mainly due to the diagnosis at late disease stages, which makes these therapies ineffective (Roxburgh and Evans, 2008; Marrero et al., 2018). Because of the limited number of available drugs in HCC treatment, systematic chemotherapies generally remain the only option (Rimassa et al., 2019). Although, these chemotherapeutic agents can extend the overall patients’ survival for months, they are associated with considerable toxicity and drug resistance (Rehman et al., 2021). Thus, there is an urgent need to develop novel therapeutic drugs with tolerable toxicity or minimal adverse effects on the patients.

Doxorubicin (DOX), is an anthracycline antibiotic and a commonly used chemotherapeutic agent to treat various solid and metastatic tumors, including HCC (Cox and Weinman, 2016; Sritharan and Sivalingam, 2021). However, clinical use of DOX is limited due to its widely reported dose-dependent and irreversible myocardial toxicity (Wu et al., 2022). DOX treated cancer survivors manifested a significantly high risk of congestive heart failure and increased death rate than the cancer survivors from cardiovascular diseases (Sheibani et al., 2022). Similarly, cisplatin (DDP) is another generic chemotherapeutic drug used to treat a wide range of solid cancers and hematological malignancies (Zhang et al., 2021). However, organ toxicity and drug resistance are the two major obstacles to its clinical use (Ghosh, 2019; Imatsuji et al., 2024). Bioaccumulation of DDP in various organs of patients induces various side effects, including nephrotoxicity, hepatotoxicity, neurotoxicity, and cardiotoxicity (Dasari et al., 2022).

β-caryophyllene (BCP) is a bicyclic sesquiterpene extracted from several plants, including black pepper, hops, copaiba, rosemary, cloves, and numerous essential oils, and is traditionally used as a food additive and multipurpose therapeutic agent (Fidyt et al., 2016; Yoo and Jwa, 2018). BCP is the first cannabis-derived compound identified as a selective agonist of the cannabinoid 2 (CB2) receptor (Hartsel et al., 2016). In preclinical studies, BCP is associated with a wide range of salutary activities, including antioxidant, anti-inflammatory, and protective actions (Francomano et al., 2019). A study by Chen et al. demonstrated the neuroprotective role of BCP through enhancing the binding of CI-AMPARs to the synaptic membrane and subsequently stimulating the cAMP/PKA pathway. The above mechanism is essential for the prevention of acute and post-acute ischemic stroke (Chen et al., 2020). Recently, BCP has been reported to suppress the growth and proliferation of cancer cells, and induce their apoptosis and cell cycle arrest, including ovarian, lung, colorectal, breast, oral, skin, and liver cancers as well as leukemia cells (Mancinelli et al., 2017; Pavithra et al., 2018; Mboge et al., 2019; Arul et al., 2020; Dahham et al., 2021; Gu et al., 2021; Ahmed et al., 2022; Ramachandhiran et al., 2022). BCP isolated from Commiphora gileadensis has been demonstrated to possess selective anticancer activity against murine lymphoma cells through inducing DNA damage, caspase 3 and eventually apoptosis (Amiel et al., 2012). Furthermore, BCP has been implicated in inhibiting the proliferation and enhancing DOX-sensitivity of cholangiocarcinoma cells and preventing drug toxicity in normal cholangiocytes (Di Sotto et al., 2020).

Although the inhibitory and antiproliferative activities of BCP against various malignancies have been reported, the critical pathways and essential genes targeted by BCP have not been extensively investigated. In the current study, we investigated the anticancer effects of BCP against HCC cells and examined the effects of its combinations with DOX and DDP on both HCC and normal liver cells. RNA-sequencing (RNA-seq) analysis was performed to investigate differentially expressed genes (DEGs) between BCP- and control-treated HCC HepG2 cells. Using a variety of informatic approaches, we conducted an extensive investigation to identify key genes and signaling pathways regulated by BCP to exert its antiproliferative activity. Subsequently, the obtained data were integrated to align the DEGs of BCP-treated HepG2 cells with a The Cancer Genome Atlas (TCGA) dataset for further verification.

## 2 Materials and methods

### 2.1 Cell culture and reagents

HCC cell lines HepG2, SMMC-7721, and human hepatocyte cell line HL-7702 were purchased from the Institute of Biochemistry and Cell Biology (Shanghai, China). HepG2 was cultured in MEM medium, while SMMC-7721 and HL-7702 were cultured in RPMI-1640 medium; both media were supplemented with 10% fetal bovine serum (FBS) and 1% penicillin/streptomycin at 37°C in an atmosphere with 5% CO_2_. BCP (≥99.12%; cat# HY-N1415), DOX (≥99.58%; cat# HY-15142), and DDP (≥99.70%; cat# HY-17394) and anti-Flag (cat# F1804) antibody were purchased from Sigma-Aldrich (St. Louis, MO). Antibodies for PARP (cat# 9542P), cleaved PARP (cat# 5625S), and caspase 3 (cat# 9662S) were purchased from Cell Signaling Technology, Inc. (Danvers, MA). Affinity Biosciences (cat# AF7022) and (cat# AF7018) antibodies were used against cleaved caspase 3 and β-actin, respectively.

### 2.2 Cell viability assay

Cell viability assay using the CCK8 reagent (Bimake, Shanghai, China) was conducted according to the manufacturer’s instructions. The cell proliferation data were used to calculate the cell viability. Briefly, HepG2, SMMC-7721, and HL-7702 cells with a density of 5 × 10^3^ cells/well were seeded into each well of 96-well plates and incubated overnight. BCP stock solution (400 mM) was prepared in ethanol, and DOX and DDP stock solutions (20 mM) were individually prepared in DMSO. In all treatments, ethanol and DMSO were used as vehicle controls, and their addition to the culture medium did not exceed 0.1% (v/v) or 0.5% (v/v), respectively. The dosages of DOX and DDP were previously determined (Jehan et al., 2020; Farooq et al., 2023). Gradient concentrations used in all treatments were 25, 50, 100, 200, 300, 400, 600, and 800 µM for BCP in combination with DOX (0.25, 0.5, 1, 2, 4, and 8 µM) and DDP (2.5, 5, 10, 20, 40, and 80 µM) prepared using the cell culture media to treat the cells in triplicates. After 48 h, 10 µL of CCK8 reagent was added to each well, followed by 2 h incubation at 37°C and measurement of absorbance at 450 nm using a microplate reader (Molecular Devices, LLC.). The cell viability percentage of all compounds or their combinations at certain concentrations during the treatment period was evaluated by normalizing absorbance at 450 nm to that of vehicle controls. The half maximal inhibitory concentrations (IC_50_) values were calculated using the GraphPad Prism 8 software.

### 2.3 Transwell assay

The transwell migration and invasion experiments were conducted using a 24-well plate (cat# 3422, Corning). HepG2 and SMMC-7721 cells (5 × 10^4^) were suspended in 100 µL of medium supplied with ethanol, 150 and 300 µM of BCP and added to the top chamber, while 600 µL of complete medium containing 10% FBS was added into the lower chamber. After 48 h of incubation at 37°C, the cells from the top chamber were carefully removed using a cotton swab, while the cells penetrated through the membrane were fixed using 600 µL of 70% methanol for 15 min. To stain the cells, 600 µL of 0.2% crystal violet was used to treat the Transwell inserts for 10 min, followed by washing and drying, and the membranes of the inserts were photographed using an inverted microscope (Leica DM13000 B). The positive staining area was calculated using ImageJ software.

For invasion assay, Matrigel (Corning, cat# 356234) was used to precoat the top chamber. Cells (8 × 10^4^) were added to the top chamber and the following procedure was the same as the migration assay described above.

### 2.4 Evaluating the phenotypic effects of combined treatments

The antiproliferative effects of BCP in combination with DOX and DDP were evaluated by the combination index (CI) using the formula: CI = (D)_1_/(Dx)_1_ + (D)_2_/(Dx)_2_. In this formula, (D)_1_ and (D)_2_ represent the doses of BCP and DOX (or DDP), respectively, when combinatorially used to achieve a specific effect (e.g., 50% cell growth inhibition). (Dx)_1_ and (Dx)_2_ are the doses of BCP and DOX (or DDP), respectively, required to achieve a similar inhibitory effect when used alone (Chou et al., 1993). The effects of the combinatorial inhibition were visualized by isobologram analyses (Tallarida, 2001). The IC_50_ values of each cell line were arranged for the isobologram analysis so that the IC_50_ of DOX represents a point on the X-axis and the IC_50_ of BCP represents a point on the Y-axis. A line was drawn to link the two IC_50_ points. Each dot in the isobologram represented an IC_50_ value of a BCP and DOX combination. The dots above, on, or below the line indicate the “antagonistic,” “additive” and “synergistic” effects, respectively. The same method was used to obtain the CI value for the cotreatment of BCP and DDP.

### 2.5 Cell apoptosis assay

The cell apoptosis assay was performed as previously described (Zhong et al., 2019; Farooq et al., 2023). The cells grown overnight in 12-well plates were treated alone by BCP, DOX, and DDP or in their combinations, while ethanol was used as a control. After 48 h of treatments, cells were collected by trypsinization, washed twice with pre-cold PBS, and stained for 10 min using 10 μg/mL of Annexin V-FITC and 10 μg/mL of propidium iodide (PI) using Annexin V-FITC Apoptosis Detection Kit (cat# A211-02, Vazyme Biotech Co. Ltd., Nanjing, China). The cells were analyzed using flow cytometer (BECKMAN COULTER, cytoFLEX, United States).

### 2.6 Western blot analysis

HepG2, SMMC-7721, and HL-7702 cells were seeded into a 6 cm plate with a density of 5 × 10^5^ and treated with the compounds for 48 h, washed with ice-cold PBS, and lysed in protein lysate buffer containing 50 mM Tris pH 7.5, 5 mM EDTA, 0.1% NP40, 300 mM NaCl, and 1× protease inhibitor. Protein concentration was calculated using the Bradford protein assay. Each protein samples (20 µg) were resolved by SDS-PAGE and transferred to poly-vinylidene difluoride transfer (PVDF) membranes then blocked for 1 h at room temperature using 5% skim milk in TBST, followed by overnight incubation with a specific primary antibody at 4°C. After three washes with TBST, membranes were incubated with a secondary antibody (1:5,000) for 1 h and the immunoblot signals were visualized using enhanced chemiluminescence (ECL) kit (cat#E411-04, Tanon, Shanghai, China).

### 2.7 RNA isolation, transcriptome analysis and quantitative RT- PCR

HepG2 cells were treated by 150 µM BCP or ethanol for 24 h, followed by total RNA extraction using the TRIzol reagent (Thermo Fisher Scientific, cat#15596026). The quality and concentration of RNA were examined by MOPS RNA electrophoresis and Eppendorf BioSpectrometer. The transcriptome analysis was performed by BGI-Wuhan China. The RNA-seq dataset of this study is accessible in the Gene Expression Omnibus (GEO) with an access number of GSE276099. The sequence library for each sample was prepared using the BGISEQ-500 platform with a 50-bp single-end (SE50) format. HISAT (v2.2.1) was used to align the clean reads to the reference genome and alignment of the clean reads to the reference genes was carried out using Bowtie2 (v2.4.5) (Langmead and Salzberg, 2012; Kim et al., 2015). Gene expression levels were calculated by RSEM (v1.3.1) (Li and Dewey, 2011). Analysis of differential gene expression was performed using edgeR in the Bioconductor software package (Robinson et al., 2010). Using a standard protocol, the RNA samples were also examined by reverse transcription and quantitative polymerase chain reaction (RT-qPCR). In brief, for reverse transcription, total RNA (1 μg) and gDNA wiper Mix were incubated at 42°C for 2 min. The samples were immediately incubated with a master mix containing 10 pmol/μL of poly (dT) primers at 37°C for 15 min and 85°C for 5 s. To detect the expression of specific genes by qPCR analysis, gene-specific primers (shown in Supplementary Table S1) were mixed with the ChamQ Universal SYBER qPCR Master Mix and the reaction was conducted using the Roche Lightcycler 480. The GAPDH value was used to normalize the results of each gene.

### 2.8 KEGG and GO enrichment analyses

The RNA-seq data of BCP-treated HepG2 cells were screened for DEGs. Gene symbols were turned into Enterz ID using the org.Hs.eg.db Bio-conductor R package. The cluster profiler (v4.6.2) package was used to perform the Kyoto Encyclopedia of Genes and Genomes (KEGG) and Gene Ontology (GO) enrichment analyses (Wu et al., 2021). Following the Transcript per million (TPM) data of the dataset, the line chart, and heatmap were plotted using ggplot2 and pheatmap R packages, respectively.

### 2.9 Survival analysis

The survival data of 371 patients were obtained from the clinical file of the TCGA-LIHC dataset, and the gene expression profiles were processed using TPM correction and then combined. To calculate the data using the R survival package, the patients’ data were separated into two groups (high and low gene expression levels) based on the mean value, and the calculated *p* values for these genes were used to generate survival curves. The Cox multiple factor analysis was carried out using the coxph function and the risk score was predicted. Subsequently, patients were split into high- or low-risk groups according to the mean risk score and the survival curves were generated. The survivalROC package in the R software was used to determine the Area Under the Curve (AUC) and the Receiver Operating Characteristic (ROC) of the RISK.

### 2.10 Lentiviral production, viral infection and ectopic PGF expression

The placental growth factor (PGF) coding sequence was amplified using gene specific primers and then subcloned into a lentiviral vector pSL4-Flag expressing the puromycin resistance gene to generate pSL4-Flag-PGF vector with a CMV promoter driving PGF expression. The primers used to amplify the PGF coding sequence, check the amplified DNA fragment and verify its ectopic expression are listed in Supplementary Table S1. Lentiviruses carrying pSL4-Flag-PGF and an empty pSL4-Flag vector were produced and used to infect HCC cells based on our previously reported procedure (Wan et al., 2012). Ectopic PGF expression was achieved by infecting HCC cells with pSL4-Flag-PGF lentivirus, and the control cells were produced by infecting HCC cells using lentivirus carrying the empty pSL4-Flag-vector.

### 2.11 Colony formation assay

HepG2 cells were cultured overnight into 12-well plates with a density of 1×10^3^ cells/well. The cells were then individually infected by pSL4-Flag-vector and pSL4-Flag-PGF lentiviruses. After 24 h, the cells were cultured in a complete medium containing 150 µM of BCP or an equal volume of ethanol for 10 days. The plates were washed using ice-cold PBS three times and cells were fixed using 70% ice-cold methanol for 20 min. To visualize the cells, 0.1% crystal violet was added in each well and incubated for 10 min, followed by extensive PBS washes. The stained wells were photographed and clonogenicity was analyzed using ImageJ software.

### 2.12 Statistical analysis

All the experiments were conducted in triplicates and/or repeated for three times, and the data were presented as mean ± standard deviation. The transcriptome data analysis was performed using the R package (v4.2.3). A student t-test and one-way ANOVA were performed for the quantitative analysis, using GraphPad Prism 8.0 (GraphPad, San Deigo, CA). A *p* value <0.05 was considered as significant. To represent the statistically significant difference, asterisks (*p < 0.05, **p < 0.01, ***p < 0.001, and ****p < 0.0001) were used to indicate different levels of significance.

## 3 Results

### 3.1 BCP exhibited relatively high inhibition to HCC cells compared to normal liver cells

**FIGURE 1:**
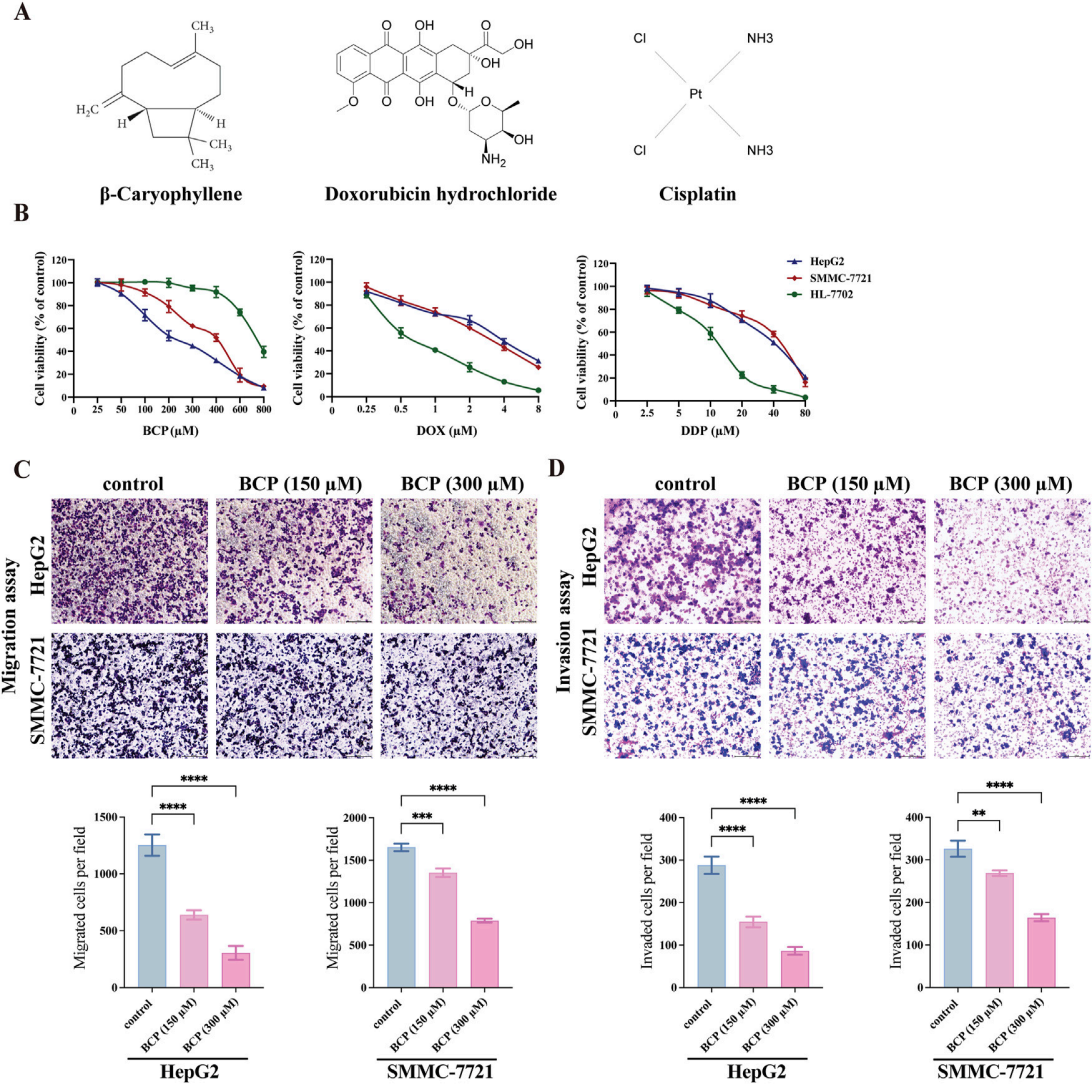
Determining the viability of HCC and normal liver cells treated by BCP, DOX and DDP. **(A)** Molecular structures of BCP, DOX and DDP. **(B)** Viability curves of HCC HepG2 and SMMC-7721 cells and normal liver HL-7702 cells after individual treatments by BCP, DOX and DDP at gradient concentrations for 48 h, followed by CCK8 assays to examine cell proliferation and calculation of cell viability. **(C)** Transwell migration, and **(D)** Matrigel invasion assays, to detect cell migration and invasion ability of HepG2 and SMMC-7721 cells treated by, 150 and 300 µM of BCP or an equal volume of ethanol for 48 h. The number of migrated or invaded cells in each chamber was assessed using crystal violet staining. Cells that penetrated the Transwell membranes were stained by crystal violet and counted under a light microscope (magnification, ×100). The values of three independent experiments were presented as mean ± standard deviation (SD). *p < 0.05, **p < 0.01, ***p < 0.001.

To determine the effects of different compounds on human HCC cells, we treated HepG2 and SMMC-7721 cells, with normal liver HL-7702 cells for comparison, for 48 h using the medium containing different concentrations of BCP, DOX, and DDP (Figure 1A), followed by CCK8 assays to examine cell proliferation. All three compounds decreased the viability of HCC cells in a dose-dependent manner (Figure 1B). BCP reduced HepG2 and SMMC-7721 cell proliferation with IC_50_ values of 193.60 ± 3.20 and 334.50 ± 2.96 μM, respectively; however, it showed low cytotoxicity to HL-7702 cells based on its relatively high IC_50_ value of 610.91 ± 2.72 μM. In contrast, DOX and DDP displayed IC_50_ values of 0.82 ± 0.09 and 11.50 ± 0.82 μM, respectively, in HL-7702 cells, but their IC_50_ values in HCC cells were relatively high in HCC cells (DOX: 1.99 ± 0.20 and 1.76 ± 0.13 μM; DDP: 27.37 ± 2.59 and 33.7 ± 1.43 μM, in HepG2 and SMMC-7721 cells, respectively) (Figure 1B; Table 1). Overall, the data indicated that DOX and DDP exhibited relatively high cytotoxicity in normal liver cells, whereas BCP displayed selective inhibition against HCC cells.

**TABLE 1:** The IC_50_ (half maximal inhibitory concentration) values of β-caryophyllene (BCP), doxorubicin (DOX) and cisplatin (DDP) individual or combinatorial treatments in HepG2, SMMC-7721 and HL-7702 cells for 48 h.

Treatment group		HepG2	SMMC-7721	HL-7702
BCP		193.60 ± 3.20	334.50 ± 2.96	610.91 ± 2.72
DOX		1.99 ± 0.20	1.76 ± 0.13	0.82 ± 0.09
DDP		27.37 ± 2.59	33.7 ± 1.43	11.50 ± 0.82
BCP (μM)				
DOX	150	0.15 ± 0.01	0.36 ± 0.03	0.66 ± 0.02
	100	0.27 ± 0.02	0.45 ± 0.04	0.71 ± 0.01
	75	0.34 ± 0.03	0.48 ± 0.02	0.73 ± 0.03
	50	0.64 ± 0.03	0.67 ± 0.08	0.74 ± 0.01
	25	0.79 ± 0.02	0.72 ± 0.01	0.81 ± 0.04
DDP	150	2.75 ± 0.32	4.57 ± 0.22	7.87 ± 0.15
	100	4.37 ± 0.43	7.23 ± 0.49	8.5 ± 0.21
	75	7.59 ± 0.91	9.87 ± 0.88	10.45 ± 0.18
	50	12.40 ± 0.60	14.11 ± 0.78	10.21 ± 0.29
	25	16.72 ± 0.37	17.22 ± 0.17	11.67 ± 0.32

### 3.2 BCP reduced migration and invasion of HCC cells

We examined the effects of BCP on HepG2 and SMMC-7721 cell migration and invasion using Transwell assays. Compared to the control using an equal volume of ethanol, HepG2 cells treated with 150 and 300 µM of BCP inhibited the cell migration by 49.01% and 75.58%, respectively. Similarly, the media containing 150 and 300 µM of BCP also reduced the migration of SMMC-7721 cells by 18.06% and 52.19%, respectively (Figure 1C). Additionally, Matrigel transwell invasion assays showed that treatments of 150 and 300 µM BCP decreased Matrigel infiltration of HepG2 cells (by 46.3% and 70.03%, respectively) and SMMC-7721 cells (by 17.67% and 49.64%, respectively) as compared to the control (Figure 1D). These results suggested that BCP could inhibit the migration and invasion of HCC cells in a dose-dependent manner.

### 3.3 BCP synergistically promoted the inhibitory activity of DOX and DDP in HCC cells

**FIGURE 2:**
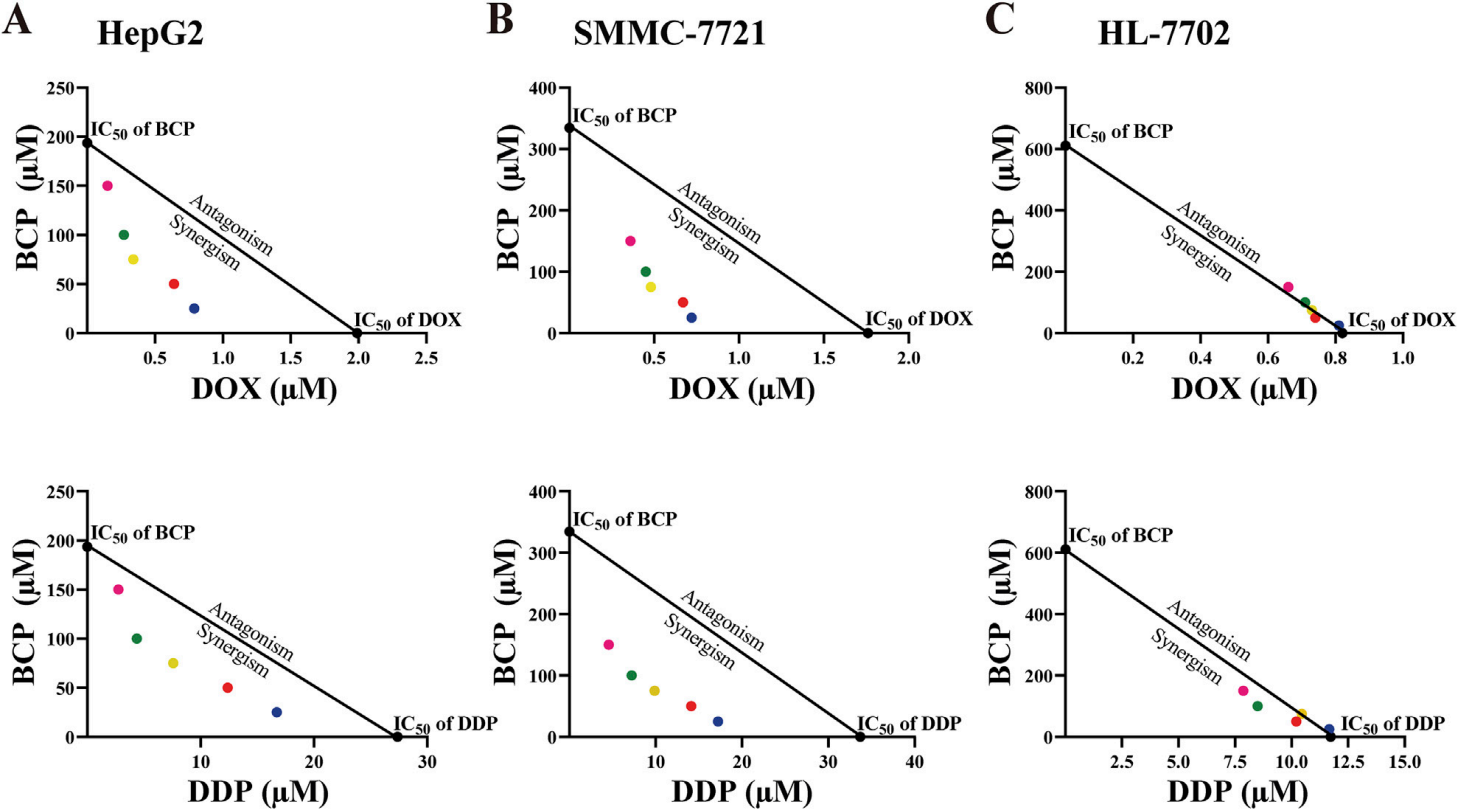
Isobologram analyses of the inhibitory effects of BCP, DOX, DDP, and their combinatorial treatments on HepG2 **(A)**, SMMC-7721 **(B)**, and HL-7702 **(C)** cells based on their IC_50_ values. Each color of the dots represents a particular BCP concentration (pink: 150 µM, green: 100 µM, yellow: 75 µM, red: 50 µM, blue: 25 µM).

To investigate potential therapeutic approaches for future clinical applications, we next determined whether BCP could improve the efficacy of DOX and DDP in eliminating HCC cells. We designed different combinations of BCP with DOX and DDP in their subtoxic concentrations, and examined their effects on HepG2, SMMC-7721, and HL-7702 cells (Figures 2A–C; Table 1). The cotreatments of 150 μM BCP with different concentrations of DOX and DDP displayed IC_50_ values of 0.15 ± 0.01 and 2.75 ± 0.32 μM in HepG2 cells and 0.36 ± 0.03 and 4.57 ± 0.22 μM in SMMC-7721 cells, respectively (Table 1), which were over 9-fold lower than the IC_50_ values in the treatments of DOX or DDP alone. Whereas, the same combinatorial treatments of BCP in HL-7702 cells showed IC_50_ values of 0.66 ± 0.02 and 7.87 ± 0.15 μM with DOX and DDP, respectively, which were only less than 1.5-fold reduction from their individual treatments. The results indicated that BCP could significantly improve the inhibitory efficacy of DOX and DDP against HCC cells, with considerably less adverse effects on HL-7702 cells.

To evaluate the possible synergism of BCP with DOX and DDP, we conducted isobologram analyses to calculate the IC_50_ values for various treatment combinations. For this purpose, we used constant BCP concentrations (25, 50, 75, 100, or 150 μM) to individually combine with a series of DOX and DDP doses. The IC_50_ data in HepG2 and SMMC-7721 cells exhibited a consistent pattern, as depicted by the dots plotted below the lines (Figures 2A, B). The data indicated that the combinations of BCP with either DOX or DDP could produce synergistic inhibition in HCC cells. However, in HL-7702 cells, almost all of the IC_50_ data of combined treatments remained on or very close to the lines connecting the IC_50_ values of the individual drug treatments on the two axes, indicating that BCP cotreatments with DOX or DDP exhibited an additive effect on normal liver cells (Figure 2C). Consistently, based on the isobologram analyses, we generated the CI values of the combination treatments of BCP with DOX and DDP in two HCC cell lines that were significantly below 1.0, indicating that these cotreatments had clear synergism. Meanwhile, the CI values of the cotreatment in HL-7702 were close to or greater than 1.0, indicating an additive inhibitory effect (Table 2).

**TABLE 2:** The combination index (CI) values of the cotreatments of BCP with DOX and DDP in HepG2, SMMC-7721, and HL-7702 cells. The values of CI indicate additive (CI = 1), antagonistic (CI > 1), or synergistic (CI < 1) inhibition of the cotreatments to cell growth.

Treatment group		HepG2	SMMC-7721	HL-7702
BCP (μM)				
DOX	25	0.53	0.48	1.02
	50	0.58	0.53	0.98
	75	0.56	0.50	1.01
	100	0.65	0.55	1.03
	150	0.85	0.65	1.05
DDP	25	0.74	0.59	1.04
	50	0.71	0.57	0.95
	75	0.66	0.52	1.01
	100	0.68	0.51	0.89
	150	0.88	0.58	0.92

### 3.4 BCP enhanced the activity of DOX and DDP to induce HCC cell apoptosis

DOX and DDP are generic chemotherapeutics to treat cancer patients clinically; however, their notable side effects have limited their clinical applications. At high doses, these two molecules eliminate both HCC cells and normal liver cells. For this reason, after discovering the anticancer synergism of BCP in combination with DOX and DDP, we investigated whether their cotreatments could selectively inhibit HCC cell proliferation without significantly damaging normal liver cells. Thus, we designated two different (low and high) drug concentrations as DOX_L_ (0.2 µM), DOX_H_ (1.5 µM), DDP_L_ (5 µM) and DDP_H_ (30 µM), and used them to treat cells either alone or in combination with 150 µM of BCP or ethanol. Thus, overnight cultured HepG2, SMMC-7721, and HL-7702 cells were treated by BCP, DOX_L_, DOX_H_, BCP + DOX_L_, DDP_L_, DDP_H_, and BCP + DDP_L_ for 48 h, followed by assays to detect the cells at early and late stages of apoptosis using Annexin V-FITC and PI reagents, respectively. The apoptotic rates were determined by fluorescence activated cell sorting (FACS) analysis.

**FIGURE 3:**
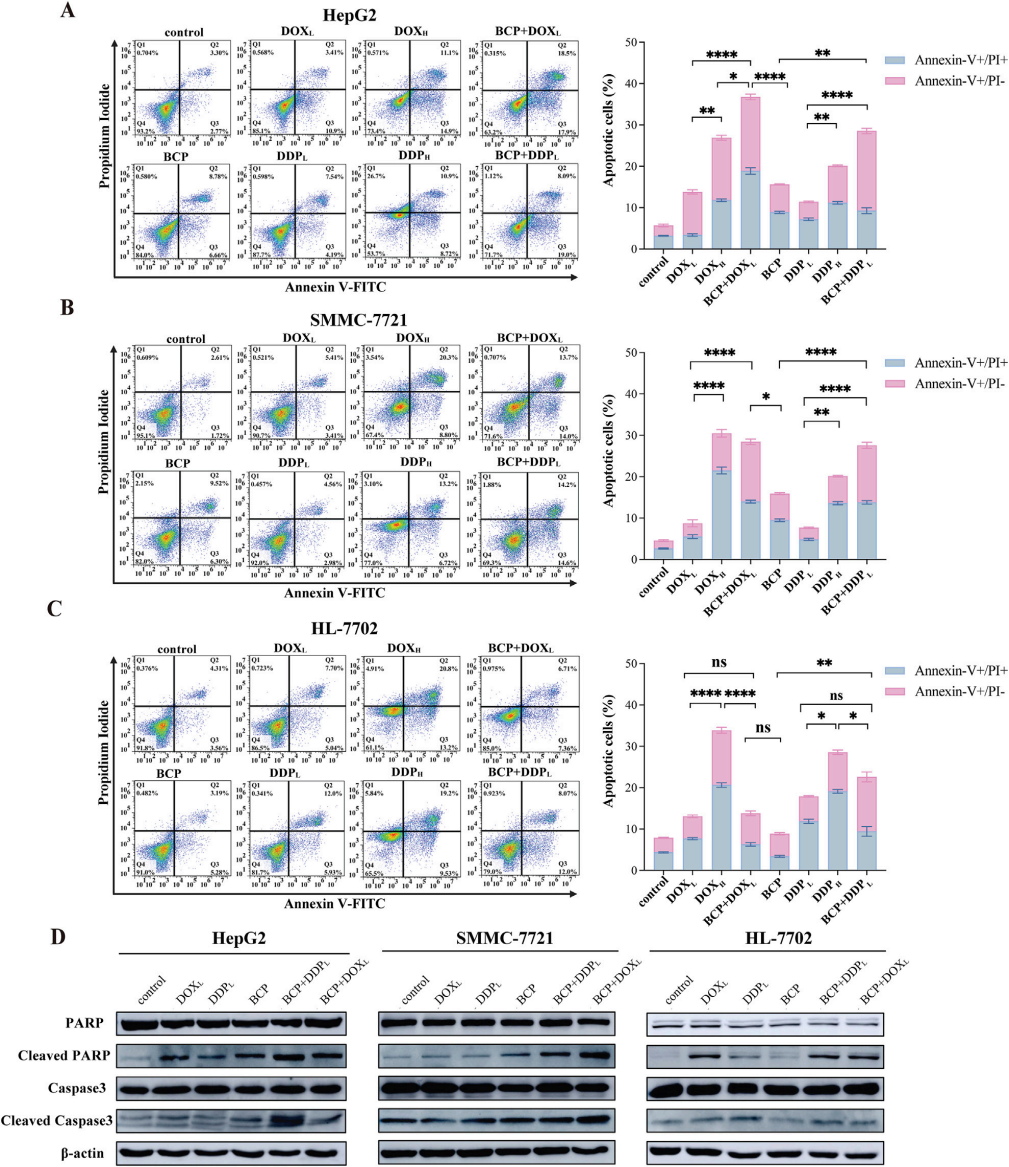
Evaluation of apoptosis in cells treated by BCP, DOX and DDP and their combinations. HepG2 **(A)**, SMMC-7721 **(B)**, and HL-7702 **(C)** cells were treated for 48 h using 150 µM BCP and its combinations with two different concentrations of DOX and DDP designated as DOX_L_ (0.2 µM), DOX_H_ (1.5 µM), DDP_L_ (5 µM), and DDP_H_ (30 µM). Cells were then stained with Annexin V-FITC and PI, and quantified by flow cytometry. Three independent experiments were conducted in triplicates and each bar represents the mean ± SD of three independent repeats. *p < 0.05, **p < 0.01, ***p < 0.001. **(D)** Western blot analysis of apoptosis-related protein in liver cells treated with chemotherapeutic agents and their combinations. Cell lysates of the above **(A–C)** treatments were examined using indicated antibodies with β-actin as a loading control. Representative images from one of the three independent experiments are shown.

In HepG2 cells, BCP + DOX_L_ or BCP + DDP_L_ cotreatments exhibited significantly increased apoptotic rates (36.77% ± 1.51% and 28.53% ± 1.35%, respectively) compared to those of DOX_L_ or DDP_L_ alone (13.40% ± 0.65% and 11.40% ± 0.52%, respectively). In SMMC-7721 cells, we observed similar results of apoptotic rates (BCP + DOX_L_ and BCP + DDP_L_: 28.44% ± 1.02% and 27.57% ± 1.14%, respectively, versus DOX_L_ and DDP_L_ alone: 8.74% ± 1.32% and 7.67% ± 0.46%, respectively) (Figures 3A, B). The data indicated that BCP could significantly improve cell apoptosis induced by DOX and DDP at their subtoxic concentrations.

To assess the potential side effects of the approach, we tested this cotreatment design on normal liver HL-7702 cells. HL-7702 cells were more sensitive to the treatments of DOX_H_ and DDP_H_ with markedly higher apoptotic rates (33.83% ± 1.27% and 28.52% ± 1%, respectively) than those of DOX_L_ and DDP_L_ alone (13.04% ± 0.57% and 17.86% ± 0.69%, respectively). However, the latter two treatments were much less effective than their combinations with BCP in inducing cell apoptosis (BCP + DOX_L_ and BCP + DDP_L_: 13.8% ± 0.98% and 22.55% ± 2.38%, respectively) (Figure 3C). These findings indicated that BCP has the potential to dramatically enhance the efficacy of low-dose DOX and DDP to induce HCC cell apoptosis with markedly low toxicity in normal liver cells, especially in the BCP + DOX_L_ combination.

We used Western blot analysis to determine the changes in apoptotic markers in liver cells treated with these DOX and DDP in the absence or presence of 150 µM BCP. When culturing HepG2 and SMMC-7721 cells in media containing BCP + DOX_L_ and BCP + DDP_L_, we observed significantly increased PARP and caspase 3 cleavage, especially the former combination in HepG2 cells and the later combination in SMMC-7721 cells, compared to DOX_L_ and DDP_L_ alone, respectively (Figure 3D). In HL-7702 cells, we detected more PARP cleavage by BCP + DDP_L_ treatment than that of DDP_L_ alone; meanwhile, BCP + DOX_L_ combination showed relatively low toxicity compared to the treatment of DOX_L_ alone (Figure 3D). Overall, these results indicated that BCP could significantly potentiate DOX- and DDP-induced apoptosis of HCC cells, but exhibit relatively low adverse effects on normal liver cells, which was generally consistent with the observations of flow cytometric studies.

### 3.5 Identification of DEGs in BCP-treated HepG2 cells

**FIGURE 4:**
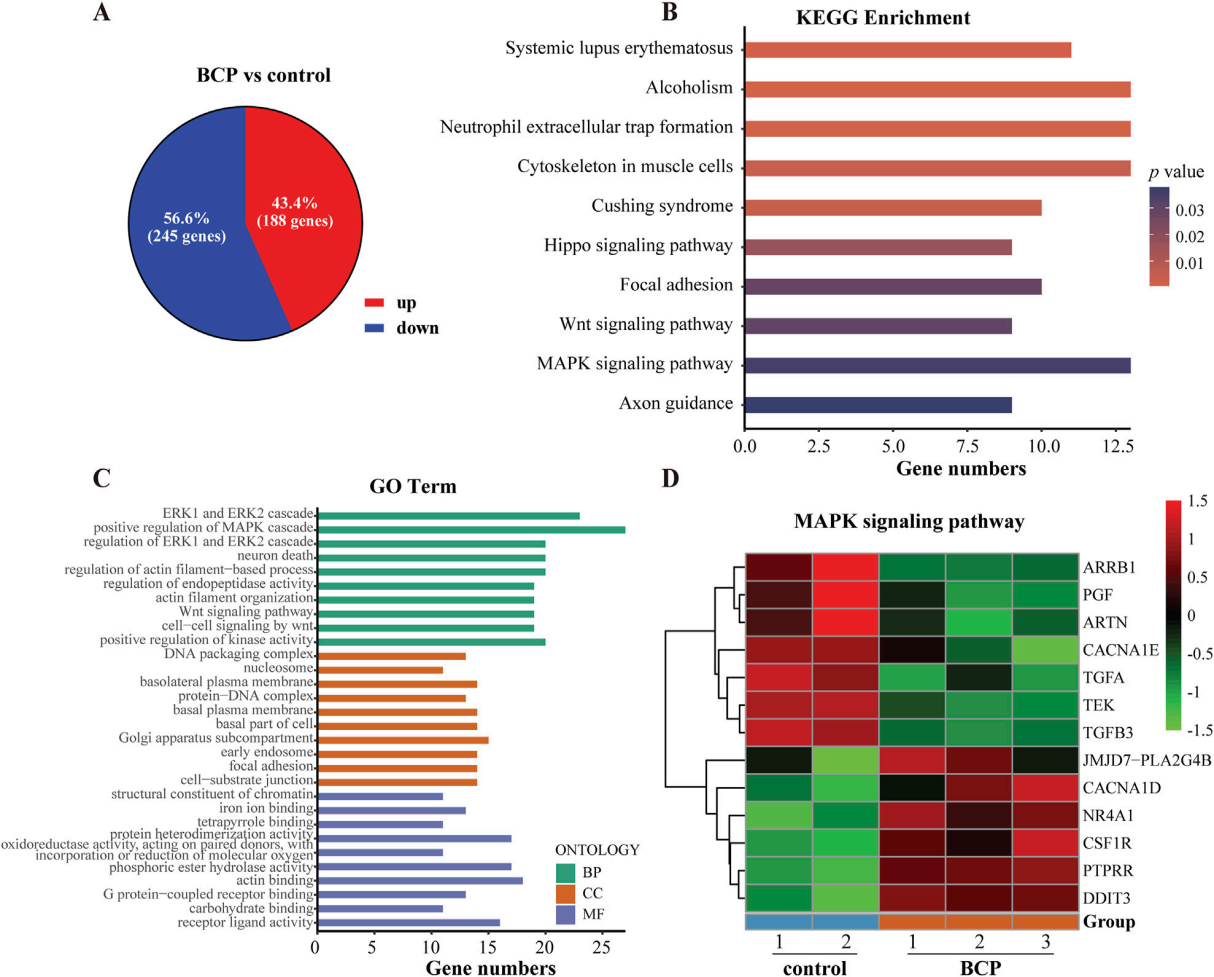
Analyses of differentially expressed genes (DEGs) using the RNA-seq data of BCP-treated HepG2 cells. **(A)** The up- and downregulated genes of BCP-treated HepG2 cells versus the control cells treated by an equal volume of ethanol. Overall, 433 DEGs were identified with 188 upregulated (red) and 245 downregulated (blue) in BCP-treated HepG2 cells compared to the control cells. **(B)** The top 10 altered signaling pathways of the KEGG enrichment analysis. **(C)** The top 10 terms of the GO enrichment analysis. **(D)** The heat map of the DEGs involved in MAPK signaling pathway in BCP-treated HepG2 cells versus the control cells.

To explore the molecular mechanism underlying the anticancer activity of BCP in HCC cells, we carried out RNA-seq analysis to determine DEGs in HCC cells treated by BCP. HepG2 cells were cultured in a medium containing 150 µM BCP for 24 h with the medium containing an equal volume of ethanol as a control, followed by extraction of total RNAs from these samples. The acquired RNA-seq data included expression of all mRNAs and other Poly(A)-containing transcripts. Thus, we first analyzed all DEGs with significantly up- or downregulated expression between BCP- and control-treated HepG2 cells. Using the DEseq2 method with a false discovery rate (FDR) ≤ 0.05 and a threshold of 1.5-fold change (FC), we discovered a total of 433 DEGs, consisting of 188 (43.4%) upregulated and 245 (56.6%) downregulated genes (Figure 4A).

To identify the mainly altered signaling pathways in BCP-treated HepG2 cells, we used the KEGG pathway enrichment to analyze these DEGs. According to the counts and *p* values, the DEGs in BCP-treated HepG2 cells were significantly enriched in the mitogen-activated protein kinase (MAPK) signaling pathway, cytoskeleton in muscle cells, Hippo signaling pathway, and Wnt signaling pathway (Figure 4B; Supplementary Table S2). Further, we employed comprehensive GO enrichment analysis to identify the specific biological processes affected by BCP. With the DEGs grouped into three GO categories to measure the functional enrichments and a *p* value threshold of ≤0.05, we discovered the most enriched top 10 GO terms from each category (Figure 4C; Supplementary Table S3). In the Biological Process (BP) category, the DEGs in BCP-treated HepG2 cells were mainly enriched in the GO terms of ERK1/ERK2 and MAPK cascades. In the Cell Component (CC) category, the DEGs were mainly enriched in the DNA packaging complex and Golgi apparatus subcompartment. In the Molecular Function (MF) category, mostly DEGs were enriched in GO terms of receptor ligand activity, structural constituent of chromatin, and iron ion binding. BCP-mediated expression changes of the genes related to the MAPK signaling pathway is shown in a heatmap analysis (Figure 4D).

### 3.6 Integrative analysis between the RNA-seq dataset of BCP-treated HepG2 cells and a TCGA-LIHC dataset

**FIGURE 5:**
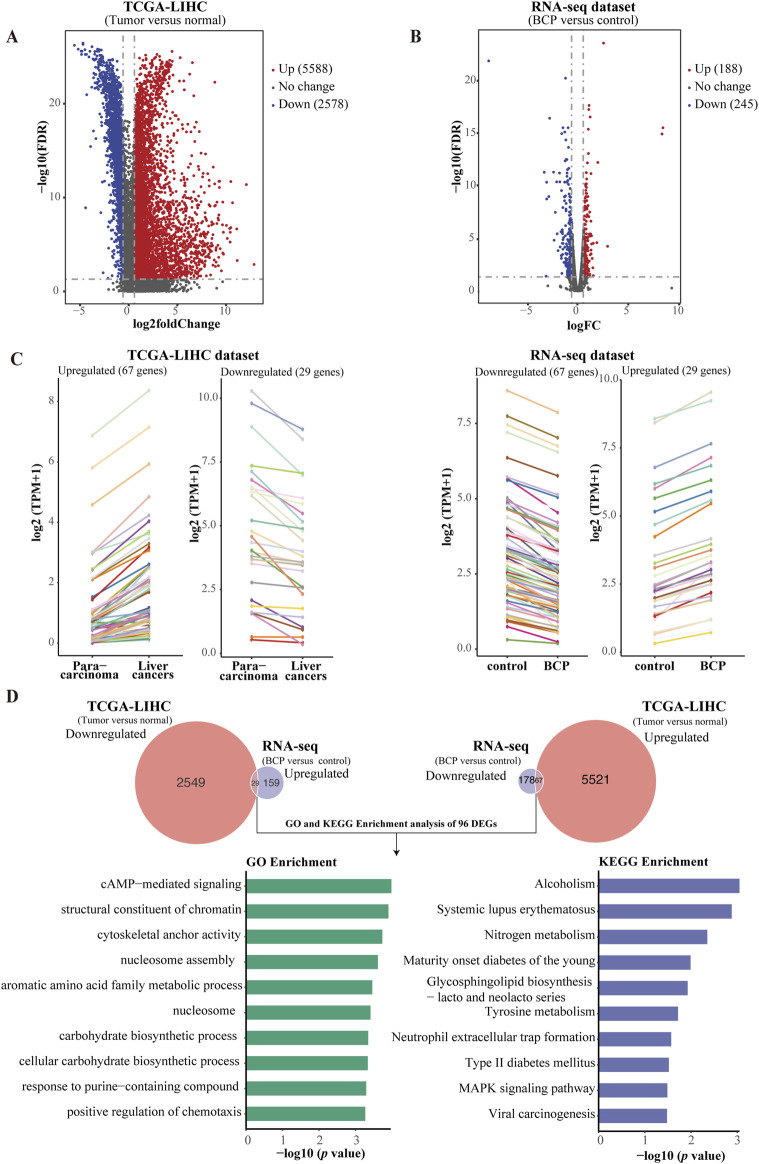
Comparative and integrative analysis using two RNA-seq datasets from TCGA-LIHC and BCP-treated HepG2 cells. **(A)** Volcano plot of the DEGs in the TCGA-LIHC dataset comprising 5,588 upregulated and 2,578 downregulated genes in HCC samples versus normal liver tissues. **(B)** Volcano plot of the BCP-treated RNA-seq dataset contains 188 upregulated and 275 downregulated genes. **(C)** Line charts depicting the genes with inverse expression patterns between the two RNA-seq datasets of TCGA-LIHC and BCP-treated HepG2 cells. In the TCGA-LIHC dataset, 67 upregulated and 29 downregulated genes were found to have inverse expression in the dataset of BCP-treated HepG2 cells. In the two datasets, each color represents the same gene. **(D)** GO and KEGG enrichment analyses of the DEGs that showed inverse expression patterns between the BCP-treated HepG2 cells and the TCGA-LIHC datasets.

Based on our RNA-seq data, BCP treatment could regulate multiple signaling pathways in HepG2 cells. Next, we asked whether BCP could affect the expression of genes that play a crucial role in the development of liver cancer. To answer this question, we conducted an integrative study by combining our RNA-seq dataset of BCP-treated HepG2 cells with a TCGA liver hepatocellular carcinoma (TCGA-LIHC) dataset established from 371 HCC samples and 50 para-carcinoma tissues (Guan et al., 2018). First, we generated two volcano plots by conducting analyses of all DEGs in liver cancer tissues versus para-carcinoma tissues from the TCGA-LIHC dataset, and similarly in BCP-treated HepG2 cells versus the control group (Figures 5A, B). In the volcano plot from the TCGA-LIHC dataset, we discovered a total of 8166 DEGs with FC > 2 and FDR <0.05, including 5588 upregulated and 2578 downregulated genes (Supplementary Table S4). The volcano plot of the RNA-seq data from BCP-treated HepG2 cells showed 433 DEGs with FC > 1.5 and FDR <0.05, including 188 upregulated and 245 downregulated genes (Supplementary Table S5). To determine if BCP treatment could alter the expression of key genes and regulatory pathways in liver cancer development, we performed integrative analyses for the DEGs of both plots with a specific focus on the genes that showed a reverse expression pattern between the two datasets. As a result, we obtained a total of 96 genes, of which 67 genes were downregulated and 29 genes were upregulated in the BCP-treated RNA-seq dataset, but showed increased or decreased expression, respectively, in the TCGA-LIHC dataset. Line charts depict the changed expression of these DEGs (Figure 5C; Supplementary Table S6). Subsequently, we used the GO and KEGG enrichment analyses to examine these genes. In the GO analysis, the top 10 enriched terms fell into the pathways related to cAMP−mediated signaling, structural constituent of chromatin, nucleosome assembly, and positive regulation of chemotaxis, among others. The most enriched top 10 KEGG pathways were mostly related to metabolism and biosynthesis, such as tyrosine metabolism, nitrogen metabolism, glycosphingolipid biosynthesis - lacto and neolacto series, and MAPK signaling pathway (Figure 5D). Hence, due to the downregulation of PGF in BCP-treated HepG2 cells and its crucial role in tumorigenesis (Bagley et al., 2011), we selected this gene for further studies.

### 3.7 Clinical prognostic values of BCP-targeted genes involved in MAPK pathway

**FIGURE 6:**
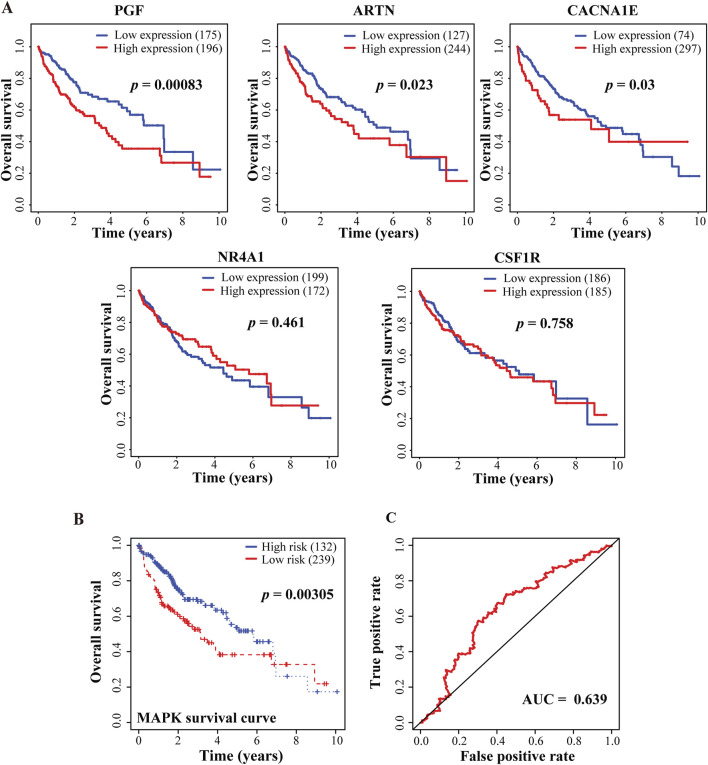
Correlative analyses between BCP-responsive regulatory DEGs and clinical outcomes of HCC patients. **(A)** Kaplan-Meier plots to evaluate the correlations of five genes involved in MAPK signaling pathway with the overall survival of the HCC patients in the TCGA-LIHC dataset. **(B)** The multivariate Cox’s regression analysis of the 3 DEGs regulating MAPK signaling pathway with the patient’s survival in the TCGA-LIHC dataset. Following the Cox’s model of these findings, the patients were divided into low- and high-risk groups (n = 239 and 132, respectively), and the Kaplan-Meier survival curves for both groups are presented. The 5-year overall survival rates were calculated as 54.8% (95% CI: 45.8%–65.9%) and 70.1% (95% CI: 63.2%–77.9%) for the high- and low-risk groups, respectively. **(C)** The receiver operating characteristic (ROC) to evaluate the accuracy of the Cox model. The value of the area under the curve (AUC) was determined as 0.639, demonstrating a high accuracy of the Cox model.

To determine the prognostic values of key genes with altered expression in BCP-treated HepG2 cells, we evaluated the 96 DEGs obtained from the integrative analyses to determine the correlations between their expression levels and clinical outcomes. Consequently, we discovered that the altered expression of 27 DEGs was significantly associated with either enhanced or decreased overall survivals of HCC patients in the TCGA-LIHC dataset (Supplementary Table S7). Among them, the expression of three upregulated MAPK signaling pathway-related genes, PGF, artemin (ARTN) and calcium voltage-gated channel subunit alpha1 E (CACNA1E), but not the two downregulated genes nuclear receptor subfamily 4 group A member 1 (NR4A1) and colony stimulating factor 1 receptor (CSF1R), are significantly associated with reduced overall survival rates of liver cancer patients (Figure 6A; Supplementary Table S8). Furthermore, for the three upregulated genes, we applied Cox’s multivariate regression analysis based on the patient profiles in the TCGA-LIHC dataset. For the obtained data, we categorized the patients into low- and high-risk groups (239 and 132 patients, respectively) using the mean risk score, and then generated the Kaplan-Meier survival curves for both groups of patients. The clinical outcomes of the low- and high-risk groups were significantly associated with the long and short-term survival rates of the patients, respectively (Figure 6B), indicating that the altered expression of these three MAPK signaling pathway-related genes has an integrative and multivariate prognostic value. Additionally, we examined our Cox model using the analysis of the receiver operating characteristic (ROC) curve, a tool to evaluate the accuracy of Cox models. The ROC analysis conducted on our data showed an AUC value of 0.639 for the 5-year overall survival (Figure 6C), which exceeded 0.5 and thus suggested high accuracy of our Cox model analysis, according to a previously reported standard (Ma et al., 2015).

### 3.8 PGF was identified as a key responsive gene to BCP-mediated inhibition in HCC cells

**FIGURE 7:**
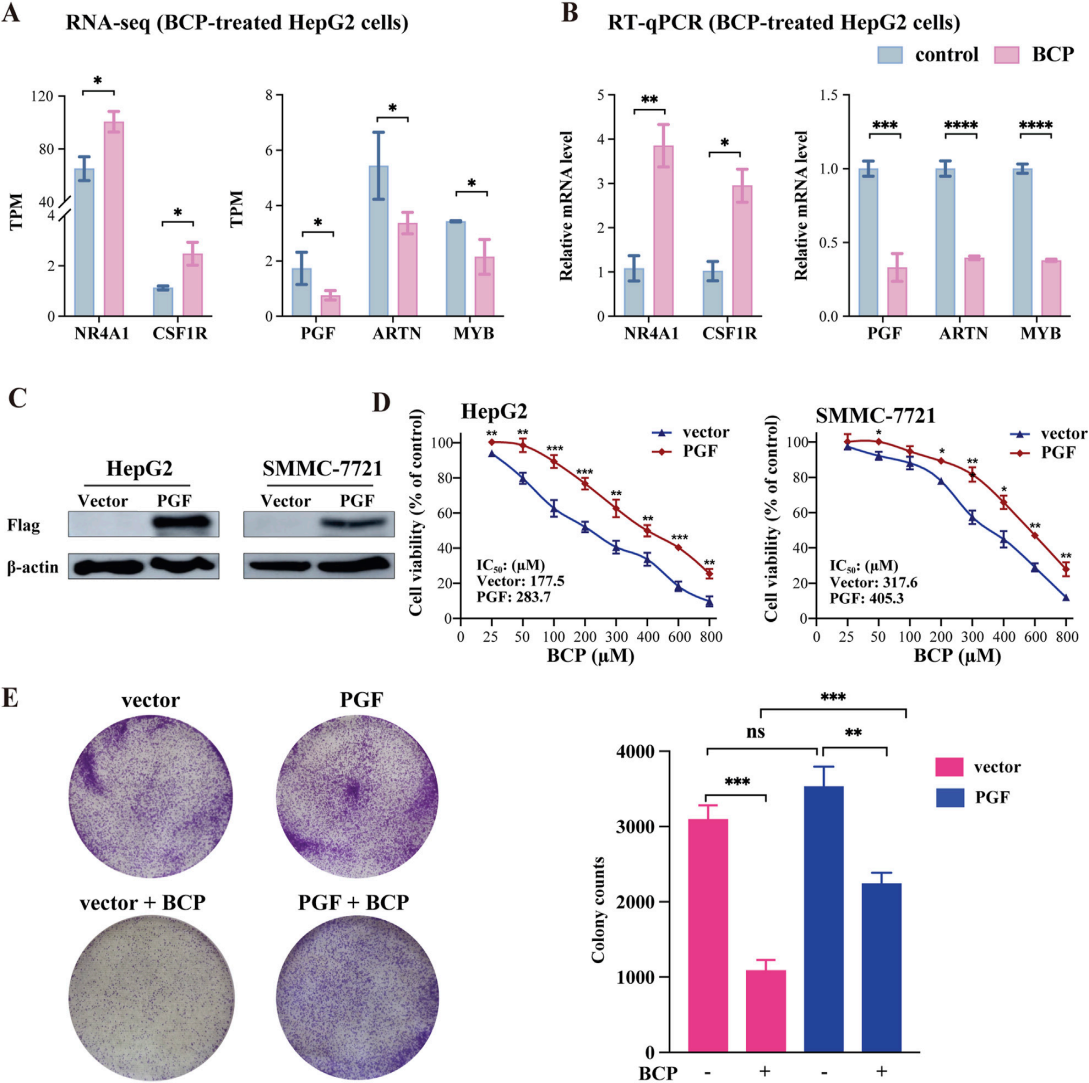
Expression analyses of BCP-mediated genes involved in MAPK signaling pathway. **(A)** TPM (transcript per million) changes of the five genes NR4A1, CSF1R, PGF, ARTN, and CACNA1E involved in the MAPK signaling pathway in the RNA-seq dataset of BCP-treated HepG2 cells versus control cells. **(B)** RT-qPCR verification of differential expression of the five genes in the BCP-treated HepG2 cells versus the control cells. **(C)** Western blot analyses to identify the ectopic PGF expression in HepG2 and SMMC-7721 cells carrying pSL4-Flag and pSL4-Flag-PGF lentiviruses with β-actin as a loading control. **(D)** Viability analysis of BCP-treated HepG2 and SMMC-7721 liver cancer cells infected by lentiviruses containing the empty pSL4-Flag vector or pSL4-Flag-PGF. Following puromycin selection, the cells were cultured in a medium with different BCP concentrations. The graph shows the cell viability calculated by measuring the cell proliferation using the CCK8 reagent. **(E)** Colony formation assay of BCP-treated HepG2 cells infected by lentivirus carrying an empty pSL4-Flag vector or pSL4-Flag-PGF. The infected cells were cultured in a medium with or without 150 µM of BCP for 10 days, followed by crystal violet staining. The graph at right represents the quantification of the colonies. *p < 0.05, **p < 0.01, ***p < 0.001.

The integrative and comparative analysis of DEGs between two datasets showed significantly altered expression of five genes that regulate MAPK signaling pathway among them, NR4A1 and CSF1R were upregulated and PGF, ARTN and CACNA1E were markedly downregulated in response to BCP treatment (Figure 7A). These results were confirmed by RT-qPCR analysis (Figure 7B). Next, we asked whether decreased PGF expression played a crucial role in BCP-mediated inhibition of HCC cell proliferation. To answer this question, we infected HepG2 cells using a lentivirus containing pSL4-Flag empty vector or pSL4-Flag-PGF with a CMV promotor to drive PGF expression. Using Western blot analysis, we confirmed the ectopic PGF expression in HepG2 and SMMC-7721 cells (Figure 7C). The infected HepG2 cells were then treated with increasing concentrations of BCP for 48 h, followed by CCK8 assays. The cells expressing ectopic PGF generally exhibited enhanced viability as compared to the control cells carrying the empty vector. Based on the cell viability data, we determined the IC_50_ value as 283.7 µM in BCP-treated Flag-PGF expressing HepG2 cells, markedly higher than that (177.5 µM) in the cells carrying an empty vector (Figure 7D, left panel). In SMMC-7721 cells, we made similar observation. In response to BCP treatment, SMMC-7721 cells expressing Flag-PGF also exhibited a higher IC_50_ value (405.3 µM) than that of the cells carrying the empty vector (317.6 µM) (Figure 7D, right panel). The results indicated that ectopically expressed PGF could desensitize HCC cells to BCP treatment. Furthermore, we performed colony formation assay to investigate the HepG2 cells expressing ectopic PGF. In response to BCP treatment, HepG2 cells carrying pSL4-vector exhibited significantly decreased colonies compared to the control without BCP; however, cells expressing ectopic PGF produced more colonies than those with empty vector in the same BCP treatment (Figure 7E). The observations suggested that PGF introduction into HepG2 cells could reduce their sensitivity to BCP-mediated growth suppression. Overall, based on our data, PGF is a crucial target gene of BCP-mediated inhibition in HCC cells.

## 4 Discussion

In the current study, we evaluated the anticancer efficacy of BCP using liver cancer HepG2 and SMMC-7721 cells and normal liver HL-7702 cells. Our investigation demonstrated that BCP treatment showed low cytotoxicity in normal liver cells in comparison to two HCC cell lines, suggesting that BCP may selectively eliminate tumors with tolerable side effects. However, BCP exhibited a relatively low killing potency in HCC cells as compared to the generic chemotherapeutics DOX and DDP. Thus, whether BCP may act as a sensitizer in cancer therapies is a logical research direction to pursue, which may extend the future clinical applications of BCP.

A previous study demonstrated that BCP increased the myocardial CB2 receptor expression to reduce DOX-induced chronic cardiotoxicity in animal model (Meeran et al., 2019). Accordingly, we investigated whether combining BCP with subtoxic concentrations of DOX and DDP could significantly inhibit HCC cell growth while causing low toxicity to normal hepatocytes. We found that BCP could synergistically enhance the anticancer effects of DOX and DDP on HepG2 and SMMC-7721 cells. Meanwhile, their combinatorial treatments exhibited minimum toxicity to normal HL-7702 cells. Consistent with our findings, a previous study indicated that BCP promoted the sensitivity of cholangiocarcinoma cells to DOX while reduced its toxicity in normal cholangiocytes (Di Sotto et al., 2020). Furthermore, another finding demonstrated the chemosensitizing effect of BCP, where lung cancer cells treated with BCP decreased the curative dosage of DDP but significantly enhanced its chemotherapeutic activity (Ahmed et al., 2022). Thus, employing BCP as an adjuvant agent in combinatorial treatments is likely a practical and effective way to mitigate the side effects of generic cancer chemotherapeutics and has the potential to take over the need for administering high doses of DOX or DDP in cancer treatments.

Malignant behavior is a complex series of steps that occur when cancer cells from a primary tumor site start to migrate to a secondary or distal organ and tissue in the body (Bozzuto et al., 2010). Thus, we further performed transwell assays and observed activities of BCP to inhibit cell migration and invasion in a dose-dependent manner, consistent with a previous report in lung cancer cells (Dahham et al., 2021; Ahmed et al., 2022).

Several studies have reported the anticancer activity of BCP, but a mechanistic investigation is still required to improve our comprehensive understanding of BCP-targeted genes and signaling pathways. After identifying the activities of BCP to decrease viability, induce apoptosis, and inhibit migration and invasion of HCC cells, we further explored gene expression alterations using RNA-seq analysis. It is noteworthy that most genes did not show very large, but still significant, changes in HepG2 cells treated by BCP, which was likely due to relatively mild or palliative effects of BCP in suppressing HCC cells. Therefore, we used a threshold of 1.5 FC to select the DEGs in response to BCP treatment. The analyses of RNA-seq data identified 433 DEGs in BCP-treated HepG2 cells. Importantly, we revealed multiple pathways and biological processes impacted by BCP treatment, including MAPK signaling pathway and ERK1/ERK2 cascade (Figures 4B, C), which regulate cell proliferation, differentiation, and migration.

Cell proliferation and differentiation are well controlled in normal cells, but during oncogenesis, cancer cells generally display aberrantly increased proliferation caused by gene mutations and altered expression of essential regulatory genes. MAPKs are activated by different stimuli that regulate intracellular signaling cascades involved in various cellular processes, including cell proliferation, differentiation, apoptosis, and survival (Kim and Choi, 2010). The MAPK/ERK signaling pathway in HCC is regulated by various growth factors, and aberrant regulation of this pathway in cancers results in increased cell growth, survival, and metastasis (Moon and Ro, 2021).

PGF belongs to the vascular endothelial growth factor (VEGF) family. Primarily, it is involved in angiogenesis and proliferation of cancer cells by promoting their growth and metastasis (Luttun et al., 2004; Fischer et al., 2007). Importantly, PGF is highly upregulated in multiple cancers including breast cancer, colorectal cancer, and endometrial carcinoma, and its expression levels positively correlated with poor prognosis, including tumor reoccurrence and metastasis, of the patients (Parr et al., 2005; Wei et al., 2005; Coenegrachts et al., 2013). In the integrative and comparative analysis of DEGs in the dataset of BCP-treated HepG2 cells with the TCGA-LIHC dataset from liver cancer patients, we discovered BCP-mediated alterations of five MAPK signaling pathway-related genes, including upregulated NR4A1 and CSF1R and downregulated PGF, ARTN and CACNA1E. Additionally, to examine the essential role of PGF decrease in reduced cell proliferation caused by BCP treatment, we ectopically expressed PGF in HCC cells, and observed that exogenous PGF could desensitize BCP-mediated growth suppression (Figures 7C–E). The mechanism underlying the desensitization of HCC cells to BCP treatment was likely through MAPK pathway stimulation by exogenous PGF whose expression was independent of BCP, and the activated downstream signaling could prevent cell apoptosis. Consistent with our findings, a previous study showed that downregulation of PGF using anti-PGF antibodies resulted in the inhibition of tumor growth and metastasis in medulloblastoma cells (Dewerchin and Carmeliet, 2014). In another study, silencing or inhibition of PGF markedly reduced the tumor burden by suppressing the tumor blood vessel formation and reducing inflammation and metastasis in a mouse model of HCC (Heindryckx et al., 2013). Among these five genes, three of them showed a positive correlation between their expression and poor clinical prognosis in HCC patients using the univariate and multivariate analyses. Importantly, our study demonstrated that the MAPK signaling pathway is the primary target of BCP in HCC.

The current study has the following limitations. First, the biomolecules directly interacted by BCP remain unclear, which deserves future investigation to understand the pharmacological action at the molecular level and design more effective therapeutics. Second, we used two HCC cell lines to evaluate the anticancer effects of BCP. Since cancers have high heterogenicity, we cannot exclude that the anticancer effects of BCP may be dampened in other cancer cell lines with specific genetic or epigenetic alterations. Third, we cannot completely exclude the alterations of other genes or signaling pathways that contribute to the anticancer activity of BCP in HCC cells. Nevertheless, based on our RNA-seq analyses, BCP-mediated reduction of HCC cell proliferation could be the combinatorial effects of the multiple pathways, with the MAPK pathway as its primary target.

## 5 Conclusion

The current study identified that BCP dampens malignant properties of HCC cells and enhances the anticancer activities of DOX and DDP. BCP also mitigates the cytotoxic effects of these two commonly used chemotherapeutics in normal liver cells. Our RNA-seq data indicated that the BCP regulates the expression of genes involved in various signaling pathways and biological processes with the PGF gene and MAPK pathway as primary targets. Overall, this study highlighted the anticancer activity of BCP as a naturally occurring sesquiterpene, and revealed its potential as a both chemotherapeutic and chemoprotective agent in HCC therapies.

## Data Availability

The datasets presented in this study can be found in online repositories. The names of the repository/repositories and accession number(s) can be found below: https://www.ncbi.nlm.nih.gov/geo/, GSE276099.
