# Supplementary material for: β-caryophyllene sensitizes hepatocellular carcinoma cells to chemotherapeutics and inhibits cell malignancy through targeting MAPK signaling pathway
Source: Front Pharmacol. 2024 Dec 13;15:1492670. doi: 10.3389/fphar.2024.1492670 (PMC11671526; doi:10.3389/fphar.2024.1492670)
Supplement: Supplementary file 3 [file Table1.docx]

**Supplementary Table S1.** Primers or oligonucleotides used in conducting quantitative polymerase chain reaction (qPCR) and generating PGF expression vector.

| **Primer name** | **Sequences (5’-3’)** | **Purpose** |
| --- | --- | --- |
| PGF-617U20 | GGCGATGAGAATCTGCACTG | qPCR for PGF,  forward primer |
| PGF-838L19 | GTGGCAGTCTGTGGGTCTC | qPCR for PGF,  reverse primer |
| ARTN-98U20 | CTGCCAAGGCCACACTTTTG | qPCR for ARTN,  forward primer |
| ARTN-248L18 | TCCTGTTGAGGCAGCACG | qPCR for ARTN,  reverse primer |
| CACNA1E-122U19 | TTGTCTGGATGCGGCTCTG | qPCR for CACNA1E,  forward primer |
| CACNA1E-359L20 | GCTTTCGTCTGCTTGTAGGC | qPCR for CACNA1E,  reverse primer |
| NR4A1-17U20 | GCTACGAAACTTGGGGGAGT | qPCR for NR4A1,  forward primer |
| NR4A1-318L20 | CGGTGCTGGTGTCCCATATT | qPCR for NR4A1,  reverse primer |
| CSF1R-855U20 | AGCTCGCAATCCCTCAACAA | qPCR for CSF1R,  forward primer |
| CSF1R-1108L20 | GGCCTCCACCATGACTTTGA | qPCR for CSF1R,  reverse primer |
| GAPDH-355U20 | GTCAAGGCTGAGAACGGGAA | qPCR for GAPDH,  forward primer |
| GAPDH-512L20 | AAATGAGCCCCAGCCTTCTC | qPCR for GAPDH,  reverse primer |
| PGF-BamHI-4U19 | cgc GGATCC ccggtcatgaggctgttcc | Primers for amplifying the PGF coding sequence and subcloning it into pSL4 vector |
| PGF-EcoRI-513L21 | ccg GAATTC ttacctccggggaacagcatc |  |
| HMS-CMV-P-484U21 | aggcgtgtacggtgggaggtc | Primers for DNA sequencing to check the PGF coding sequence |
| HMS-IRES-58L22 | aaacgcacaccggccttattcc |  |
